# Molecular Connections between DNA Replication and Cell Death in β-Amyloid-Treated Neurons

**DOI:** 10.2174/1570159X21666230404121903

**Published:** 2023-07-10

**Authors:** Filippo Caraci, Annamaria Fidilio, Rosa Santangelo, Giuseppe Caruso, Maria Laura Giuffrida, Marianna Flora Tomasello, Ferdinando Nicoletti, Agata Copani

**Affiliations:** 1 Department of Drug and Health Sciences, University of Catania, Catania, Italy;; 2 UOR of Neuropharmacology and Translational Neurosciences, Oasi Research Institute - IRCCS, Troina, Italy;; 3 Institute of Crystallography, National Council of Research, Catania Unit, Catania, Italy;; 4 Department of Physiology and Pharmacology, University Sapienza of Rome, Rome, Italy;; 5 IRCCS Neuromed, Pozzilli, Italy

**Keywords:** ATM/ATR, Chk-1, Claspin, caspase-7, cell cycle, apoptosis, Alzheimer’s disease

## Abstract

**Background:**

Ectopic cell cycle reactivation in neurons is associated with neuronal death in Alzheimer’s disease. In cultured rodent neurons, synthetic β-amyloid (Aβ) reproduces the neuronal cell cycle re-entry observed in the Alzheimer’s brain, and blockade of the cycle prevents Aβ-induced neurodegeneration. DNA polymerase-β, whose expression is induced by Aβ, is responsible for the DNA replication process that ultimately leads to neuronal death, but the molecular mechanism(s) linking DNA replication to neuronal apoptosis are presently unknown.

**Aim:**

To explore the role of a conserved checkpoint pathway started by DNA replication stress, namely the ATM-ATR/Claspin/Chk-1 pathway, in switching the neuronal response from DNA replication to apoptosis.

**Methods:**

Experiments were carried out in cultured rat cortical neurons challenged with toxic oligomers of Aβ protein.

**Results:**

Small inhibitory molecules of ATM/ATR kinase or Chk-1 amplified Aβ-induced neuronal DNA replication and apoptosis, as they were permissive to the DNA polymerase-β activity triggered by Aβ oligomers. Claspin, *i.e*., the adaptor protein between ATM/ATR kinase and the downstream Chk-1, was present on DNA replication forks of neurons early after Aβ challenge, and decreased at times coinciding with neuronal apoptosis. The caspase-3/7 inhibitor I maintained overtime the amount of Claspin loaded on DNA replication forks and, concomitantly, reduced neuronal apoptosis by holding neurons in the S phase. Moreover, a short phosphopeptide mimicking the Chk-1-binding motif of Claspin was able to prevent Aβ-challenged neurons from entering apoptosis.

**Conclusion:**

We speculate that, in the Alzheimer’s brain, Claspin degradation by intervening factors may precipitate the death of neurons engaged into DNA replication.

## 1 INTRODUCTION

Expression of cell cycle proteins and replicative DNA synthesis have been observed in neuronal populations that eventually degenerate in the Alzheimer’s disease (AD) brain [1]. The reentry into the cell cycle of post-mitotic neurons has been associated with neuronal apoptosis also in Parkinson’s disease [2], Huntington’s disease [3], and amyotrophic lateral sclerosis (ALS) [4], leading to the hypothesis that reentering the cell cycle makes neurons prone to death. Mitosis itself has not been found, suggesting that adult neurons are prevented from entering the M phase while maintaining the DNA replication status over a long time, possibly years [5]. So far, the molecular mechanisms regulating survival and death in these “hibernating” neurons are unknown. We meant to analyse the molecular connector(s) bridging DNA replication to neuronal apoptosis, which might lead to the identification of new neuroprotective strategies. We previously showed that, in cultured rat cortical neurons, synthetic β-amyloid (Aβ) reproduces the neuronal cell cycle re-entry observed in the human AD brain, including the expression of the molecular repertoire necessary for the G1/S transition (*i.e*., cyclin D1, cyclin E, and phosphorylated retinoblastoma protein), and that blockade of cell cycle activation prevents Aβ-induced neurodegeneration [6]. We have also demonstrated that, following cell cycle reactivation in neurons, Aβ induces the overexpression of the repair enzyme DNA polymerase-β (DNA pol-β), which in neurons carries out the *de novo* DNA synthesis, ultimately resulting in apoptotic death [7-10]. In the current study, we investigate the checkpoint pathway that monitors DNA replication in proliferating cells, namely the ataxia-telangiectasia mutated (ATM) - ataxia telangiectasia and Rad3-related (ATR)/Claspin/ checkpoint kinase-1 (Chk-1) pathway [11], which is able to switch the cellular response from stalled DNA replication to apoptosis [12]. We demonstrate that this checkpoint is active in cultured differentiated neurons, where blockade of the ATM/ATR kinase or of the Chk-1 facilitated Aβ-induced DNA replication with an ensuing increase in neuronal apoptosis. We also provide indications that Claspin, a chromatin-interacting protein that bridges the ATM/ATR kinase with the Chk-1 [13], could be involved in monitoring DNA replication in Aβ-challenged neurons and propose that Claspin degradation by caspase-7 could trigger the death of neurons undergoing DNA replication. Finally, current data suggest that the inhibition of caspase-7 may provide an effective tool for preventing the activation and execution of apoptosis in neurons that have entered the S phase in response to Aβ. We show that these neurons do not seem prone to assume the potentially detrimental inflammatory phenotype described, for example, in cycling ALS neurons [14], although their functional activity remains unknown.

## 2 MATERIALS AND METHODS

### 2.1 Pure Neuronal Cultures and Treatments

Cultures of pure cortical neurons (> 99% MAP-2^+^/GFAP^-^) were obtained from rats at embryonic day 15 (E15), as described previously [6]. In brief, cortices were dissected into Ca^2+^/Mg^2+^ free buffer; dissociated cells were plated on 24-well Nunc plates precoated with 0.1 mg/ml poly-D-lysine at a density of 5 x 10^5^/dish and maintained in a chemically defined medium for 8-12 days *in vitro* (DIV). Cytosine-β-D-arabinofuranoside (10 µM) was added to the cultures 18 h after plating and kept for 4 days before medium replacement. The absence of the neuronal precursor marker, nestin, as assessed by immunoblot analysis, indicated that all MAP-2^+^ cells were fully developed neurons (Supplementary Fig. **S1**). Aβ_(1-42)_ oligomers (1 µM) were applied to differentiated neurons at 8 days DIV, in the presence of 1 µM MK-801, to avoid the contribution of endogenous glutamate to the overall neurotoxicity. Caffeine powder (C0750, Sigma-Aldrich, Merck Group, Darmstadt, Germany), ATM/ATR kinase Inhibitor (Calbiochem # 118501, IC_50_ = 200 nM - Merck Group, Darmstadt, Germany), Chk-1 Inhibitor (Calbiochem # 371957, IC_50_ = 100 nM), and caspase-3/7 Inhibitor I (Calbiochem # 218826; Caspase-3 K_i_ = 60 nM; Caspase-7 K_i_ = 150 nM) were added for 60 min before medium replacement and Aβ addition.

### 2.2 Aβ Peptide Preparation and Analysis

Synthetic human Aβ_(1-42)_ (H-1368) was obtained from Bachem Distribution Services GmbH (Bubendorf, Germany), and Aβ_(1-42)_ oligomers were prepared according to the original protocol of Klein's group [15]. Aβ_(1-42)_ monomers were prepared from HIFP-treated Aβ_(1-42)_ according to our previously published protocol [16]. For dot blot analysis, protein samples (0.5 μg of each) were spotted onto a nitrocellulose membrane. The membrane was first probed with the rabbit polyclonal anti-oligomer A11 antibody (Thermo Fisher Scientific, 1:100 – Waltham, MA, USA), and then re-probed with the mouse monoclonal antibody 6E10 (BioLegend, 1:800 – San Diego, CA, USA), which detects all forms of Aβ (Supplementary Fig. **S2A**). For western blot analysis, protein samples (7.5 μg of each) were separated by 4-12% Bis·Tris SDS-PAGE and transferred to a nitrocellulose membrane. Then, the membrane was blotted with the mouse monoclonal antibody 6E10 (Supplementary Fig. **S2B**). Membranes were incubated with IRDye^®^ 800CW goat anti-rabbit IgG or 680LT goat anti-mouse IgG secondary antibodies (1:15,000, LI-COR 926-32211 and 926-68020, respectively) for 1 hr at room temperature (RT). Hybridization signals were detected with the Odyssey^®^ Infrared Imaging System (LI-COR Biosciences, Lincoln, NE, USA).

### 2.3 Fluorescence-Activated Cell Sorting Analysis for Simultaneous Assessment of S phase and Apoptosis

Neurons were processed for fluorescence activated cell sorting (FACS) analysis as described previously [8]. DNA content and ploidy were assessed by using a Coulter FC500 flow cytometer (Beckman Coulter, Brea, CA, USA), and cell cycle distribution profiles were analyzed with the ModFit software program. Apoptotic neurons were scored from the area of hypoploid DNA preceding the G0/G1 DNA peak. A typical cell cycle distribution profile of control neurons and neurons after exposure to Aβ_(1-42)_ oligomers or Aβ_(1-42)_ monomers as a negative control is shown in Supplementary Fig. **S3**.

### 2.4 Immunostaining and Flow Cytometric Analysis

Neuronal cells were harvested with mild trypsinization and immediately fixed with 4% paraformaldehyde for 30 min at 4°C. Neurons were permeabilized with 0.1% Triton-X100 solution in phosphate-buffered saline (PBS) for 10 min on ice and blocked with 3% bovine serum albumin (BSA) solution in PBS for 30 min. Neurons were then processed for immunostaining by 2 hr incubation at 4°C with rabbit anti-Cyclin A2 (1:300; Abcam, Cambridge, UK) or rabbit anti-ChK1 (phospho S317) (1:300; Abcam, Cambridge, UK), followed by incubation for 1 h at RT with Alexa-Fluor 488-conjugated anti-rabbit secondary antibody (1:300; Invitrogen, Thermo Fisher Scientific). Positive neurons were scored either on a Coulter FC500 flow cytometer or on a CyFlow ML flow cytometer (Partec, Canterbury, Kent, UK).

### 2.5 Cross-Linking of Chromatin Proteins and Preparation of DNA/Protein Fragments

Experiments were performed as previously described [8]. Cross-linked nuclear proteins were collected by centrifugation at 750 *g* for 10 min. Collected nuclei were resuspended in 0.5 packed cell volume lysis buffer (50 mM Tris/HCl, 10 mM EDTA, 1% sodium dodecyl sulfate (SDS), and mixture protease inhibitors) and maintained at 4°C for 10 min. Then the DNA/protein complex was sheared by sonication. A total sonication time of 3 min/sample, with a Vibra Cell (Sonics & Materials, Danbury, CT) with output control setting 5, resulted in DNA fragment sizes of 1 kb. Debris was cleared by minifuge centrifugation at maximum speed for 10 min at 4°C.

### 2.6 Immunoprecipitation of DNA/Protein Complexes

DNA/protein complexes were diluted to 200 µg/ml in chromatin immunoprecipitation buffer (containing the following (in mM): 1.2 EDTA, 16.7 Tris-HCl, 167 NaCl, pH 8.1, plus 0.01% SDS, 1.1% Triton X-100) and immunoprecipitated with 10 µg/mg protein lysate of goat anti-cell division cycle 45 (anti-Cdc45; Santa Cruz Biotechnology sc-9298) or 10 µg/mg protein lysate of normal goat IgG (Santa Cruz Biotechnology sc-2028, Dallas, Texas, USA). Immunoprecipitation was performed by incubating the lysates for 2 hr at 4°C; then 20 µl of protein G Plus-agarose was added, and samples were incubated overnight at 4°C on a rotating device (10 revolutions/min). Pellets were collected by centrifugation at 1000 *g* for 5 min at 4°C, and washed three times in PBS. After a final wash, the pellets were resuspended in 25 µl of 1X electrophoresis sample buffer and boiled for 3 min. Western blot analysis was performed as previously described [8], using goat anti-Claspin (2 µg/ml; Santa Cruz Biotechnology sc-27297) or mouse anti-DNA pol-β (2 µg/ml; Thermo Fisher Scientific MA5-13899) antibodies.

### 2.7 Western Blot Analysis

Western blot analysis was performed on either total or nuclear protein fractions. Nuclear protein fractions were obtained by using Ne-PER Nuclear and Cytoplasmic Extraction Reagents following the manufacturer’s specification (Thermo Fisher Scientific 78833). Proteins were separated on NuPage^TM^ 4-12% bis-tris gel (Thermo Fisher Scientific), and transferred to nitrocellulose membranes. The membranes were blotted with the following primary antibodies: anti-cleaved Caspase-7 (1 µg/ml, AbCam, ab2323), anti-ChK1 (phospho S317) (1:300, AbCam ab38518), anti-ChK1 (1:300, AbCam ab69536), anti-Nestin (1:300, Merck MAB353), anti-Neu (1:300, Merck MAB377), anti-H3 (1:10,000, AbCam ab1791), anti-Lamin B1 (Santa Cruz, sc-365214), anti-Claspin (1:300, Santa Cruz sc-376773 or sc-27297), and anti-DNA pol-β (1:300, Thermo Fisher Scientific MA5-13899). After washing in tris-buffered saline (TBS)/Tween 20X 0.1%, membranes were incubated with IRDye^®^ 800CW goat anti-rabbit IgG or 680LT goat anti-mouse IgG secondary antibodies (1:15,000, LI-COR 926-32211 and 926-68020, respectively) for 1 hr at RT. Hybridization signals were detected with the Odyssey^®^ Infrared Imaging System (LI-COR Biosciences) and quantified by freely available ImageJ software.

### 2.8 Peptide Transfection

GenScript Biotech Corporation (Rijswijk, Netherlands) synthesized the Chk1-binding motif consensus peptide (PP) [17]. The Chariot transfection kit, containing β-galactosidase (β-gal) as a positive control for transfection, and the β-gal staining kit were purchased from Active Motif (Carlsbad, CA). Transfections of PP (500 ng/well) and β-gal (1 µg/well) were carried out in the neuronal culture medium, for 1 and 2 hr, respectively, according to the manufacturer’s protocol. The efficiency of β-gal transfection was determined by monitoring β-gal activity through the hydrolysis of X-gal, which produces a blue color in transfected cells. Blue cells were counted by light microscopy in four random fields/dish, in two separate dishes.

### 2.9 Gene Expression Analysis by Quantitative Real-Time PCR (qRT-PCR)

Total RNA from pure neuronal cultures was extracted by using the RNeasy Mini Kit (Qiagen, Hilden, Germany) according to the manufacturer’s instructions. The concentration of total RNA recovered from each sample was determined by measuring the absorbance at 260 nm with a Varioskan^®^ Flash spectrophotometer (Thermo Fisher Scientific). SuperScript III First-Strand Synthesis SuperMix kit (Thermo Fisher Scientific) was used to carry out the reverse transcription and obtain the cDNA. All the obtained cDNA samples (100 ng), mixed with the appropriate amount of SYBR Green PCR Master Mix and specific primers (Qiagen), were loaded in a 384-well plate, and the amplification was measured through a LightCycler^®^ 480 System (Roche Molecular Systems, Inc., Pleasanton, CA, USA). The details regarding the QuantiTect Primer Assays employed for the gene expression analysis of Cxcl1 and the selected internal control, GAPDH, are the following: Cxcl1, detected transcript NM_030845, Cat. No. QT00100275; GAPDH, detected transcript NM_017008, Cat. No. QT00199633. A reaction in the absence of cDNA, representing our negative control, was performed in each run, following verification by Agilent Bioanalyzer 2100 (Santa Clara, CA, USA). The relative RNA expression level for each sample was calculated by using the 2−ΔΔCT method in which the threshold cycle (CT) value of the gene of interest was compared to the CT value obtained for the GAPDH gene.

### 2.10 Cxcl1 Secretion

Cxcl1 quantification in neuronal culture supernatants was carried out by using a Rat Cxcl1/Cinc-1 Quantikine ELISA Kit, according to manufacturer’s instructions (R&D Systems, Minneapolis, USA). Supernatants were collected, centrifuged at 1000 g for 10 min, and assayed immediately or stored at -80°C until use. Final absorbance at 450 nm was read using a Synergy H1 Hybrid Multi-Mode Microplate Reader (Biotek, Shoreline, WA, USA) within 30 min of stopping the reaction. Wavelength correction was applied by subtracting readings at 570 nm from the reading at 450 nm.

### 2.11 Statistical Analysis

Quantitative data were expressed as the mean ± standard error of the mean (SEM). Error bars were plotted only upwards in all graphs. *P* values were calculated by one-way analysis of variance (ANOVA), followed by the Holm-Sidak test for pairwise comparisons. *P* < 0.05 was considered statistically significant. Analysis was carried out using SigmaPlot 12.5. A single statistical analysis was performed for data plotted separately in Figs. (**1A**, **B** and **3A**, **B**), which resulted from the same set of experiments.

## 3 RESULTS

### 3.1 Inhibition of the ATM/ATR Signaling Pathway Amplified Aβ-induced S Phase and Apoptosis in Cortical Neurons

**Fig. (1):**
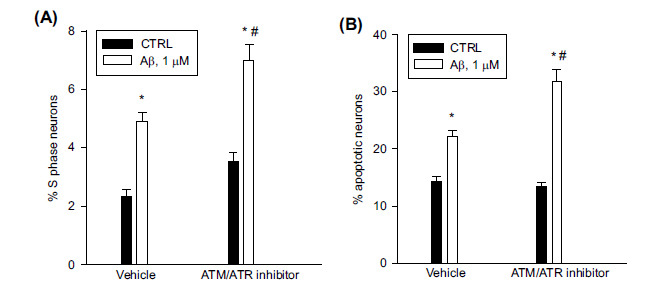
Inhibition of the ATM/ATR kinase amplified Aβ-induced S phase and apoptosis in pure cortical neurons. Pure neuronal cultures were exposed to the ATM/ATR Inhibitor (5 µM) for 1 h and then treated for 24 hr with 1 µM oligomeric Aβ_(1-42)_. S phase (**A**) and apoptotic (**B**) neurons were scored by cytofluorimetric analysis of propidium iodide (PI)-labeled samples. Values are means ± SEM of n = 24 from 6 independent experiments, the same as in Fig. (**3A-B**), in which each experimental condition was run in quadruplicate. *p < 0.05 *vs*. CTRL (control) and ^#^*p* < 0.05 *vs*. Aβ alone.

In primary pure neuronal cultures obtained from E15 rat embryos [6], we first addressed the involvement of ATM kinase in cell cycle checkpoint(s) control. A partial genetic reduction in ATM is associated with neuronal cell cycle reentry in ATM^+/-^ mice [18, 19], and a drop in ATM levels is observed in cycling neurons from the human AD brain as well as in three distinct AD mouse models [19]. Here, neurons were challenged for 1 h with a selective cell-permeable ATM/ATR kinase inhibitor (CGK-733, 5 µM), then S phase and apoptotic neurons were scored 24 hr later by FACS analysis [8]. Under these conditions, the ATM/ATR kinase inhibitor did not affect the basal number of neurons entering the S phase and apoptosis (Figs. **1A**, **B**). Then, the neuronal cell cycle was induced by exposing the cultures to 1 µM Aβ_(1-42)_ oligomers for 24 hr (Fig. **1A**). The percentage of neurons found in the S phase generally increases up to 10% from the control value between 8-16 hr after the addition of Aβ and declines by about half after 20 hr [6]. On the contrary, Aβ-induced apoptosis increases linearly with time, reflecting the transfer of S phase neurons into apoptosis [6]. Interestingly, the inhibition of the ATM/ATR kinase prior to Aβ addition amplified the Aβ-induced S phase and apoptosis at 24 hr (Figs. **1A**, **B**), supporting the hypothesis that reduced ATM signaling can contribute to neurodegeneration in AD [19]. Even caffeine, which is a direct inhibitor of the ATM kinase *in vitro* [18, 20], increased the Aβ-induced S phase and apoptosis when added at a 2 mM concentration 1 hr prior to Aβ (Supplementary Table **S1**).

**Fig. (2):**
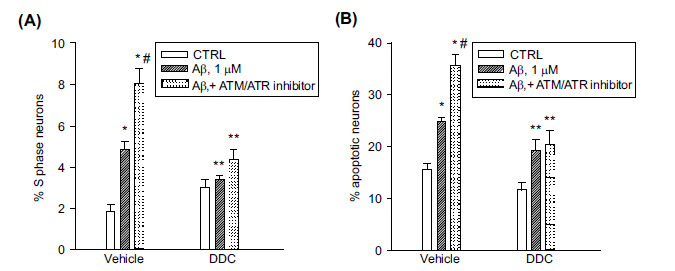
Inhibition of the ATM/ATR kinase facilitated the Aβ-induced S phase through DNA pol-β activity. Pure neuronal cultures were exposed to the ATM/ATR Inhibitor (5 µM) for 1 h and then treated for 24 hr with 1 µM oligomeric Aβ_(1-42),_ both in the presence and in the absence of dideoxycytidine (DDC, 100 µM). S phase (**A**) and apoptotic (**B**) neurons were scored by cytofluorimetric analysis of PI labeled samples. Values are means ± SEM of n = 15 from 5 independent experiments. *p < 0.05 *vs*. CTRL (control), ^#^*p <* 0.05 *vs*. Aβ alone, and **p < 0.05 *vs*. the respective conditions in the absence of DDC.

The DNA pol-β inhibitor, dideoxycytidine (DDC), is known to prevent the Aβ-induced neuronal S phase and apoptosis [7]. The addition of DDC (100 µM) to the combination of Aβ plus the ATM/ATR kinase inhibitor reduced the percentage of neurons entering the S phase and apoptosis to the same extent as the addition of DDC to Aβ alone, indicating that the inhibition of ATM/ATR signaling was permissive to the DNA pol-β activity triggered by Aβ_(1-42)_ oligomers (Figs. **2A**, **B**).

**Fig. (3):**
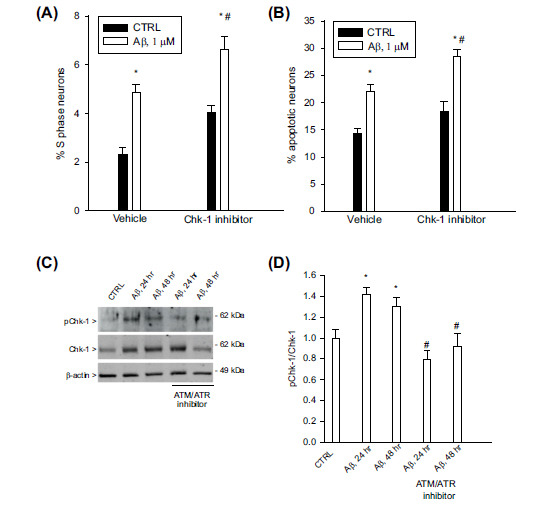
Inhibition of the Chk-1 activity amplified Aβ-induced S phase and apoptosis in pure cortical neurons. Pure neuronal cultures were exposed to the ATP-competitive inhibitor of Chk-1, isogranulatimide (500 nM for 1 h), and then treated for 24 hr with 1 µM oligomeric Aβ_(1-42)_. S phase (**A**) and apoptotic (**B**) neurons were scored by cytofluorimetric analysis of PI labeled samples. Values are means ± SEM of n = 18-24 from 6 independent experiments, the same as in Figs. (**1A**, **B**), in which the experimental conditions with the Chk-1 inhibitor were run in triplicate and the others in quadruplicate. *p < 0.05 *vs*. CTRL (controls) and ^#^ *p <* 0.05 *vs*. Aβ alone. (**C**) Representative immunoblot of total Chk-1 and phospho-Chk-1(ser 317) levels in protein extracts from cultured cortical neurons challenged with 1 µM oligomeric Aβ_(1-42)_ for 24 or 48 hr, both in the presence and in the absence of the ATM/ATR inhibitor (5 µM). The β-actin band is shown as a loading control. (**D**) Densitometric analysis of bands from three separate western blots carried out as in C. Both pChk-1and Chk-1 signals were normalized against β-actin before the calculation of the pChk-1/Chk-1 ratio (normalized to 1 in controls). Bars represent the means ± SEM of 3 determinations. *p < 0.05 *vs*. CTRL (controls) and ^#^*p <* 0.05 *vs*. Aβ alone.

The Chk-1 is the checkpoint mediator of the ATM/ATR kinase, which phosphorylates and activates Chk-1 in response to replication stress [21]. The ATP-competitive inhibitor of Chk-1, isogranulatimide (500 nM for 1 hr), mimicked the effect of the ATM/ATR kinase inhibitor, thus amplifying both S phase neurons and apoptosis (Figs. **3A**, **B**). Western blot analysis of neuronal protein extracts showed that, following Aβ exposure, the total levels of Chk-1 increased and remained high for up to 48 hr and the amount of phospho-Chk-1 (ser 317) increased in parallel (Figs. **3C**, **D**). As expected, the ATM/ATR kinase inhibitor (5 µM) prevented Chk-1 phosphorylation on the ser 317 epitope (Figs. **3C**, **D**).

### 3.2 The Adaptor Protein, Claspin, was Involved in the Aβ-induced S Phase and Apoptosis of Cortical Neurons

**Fig. (4):**
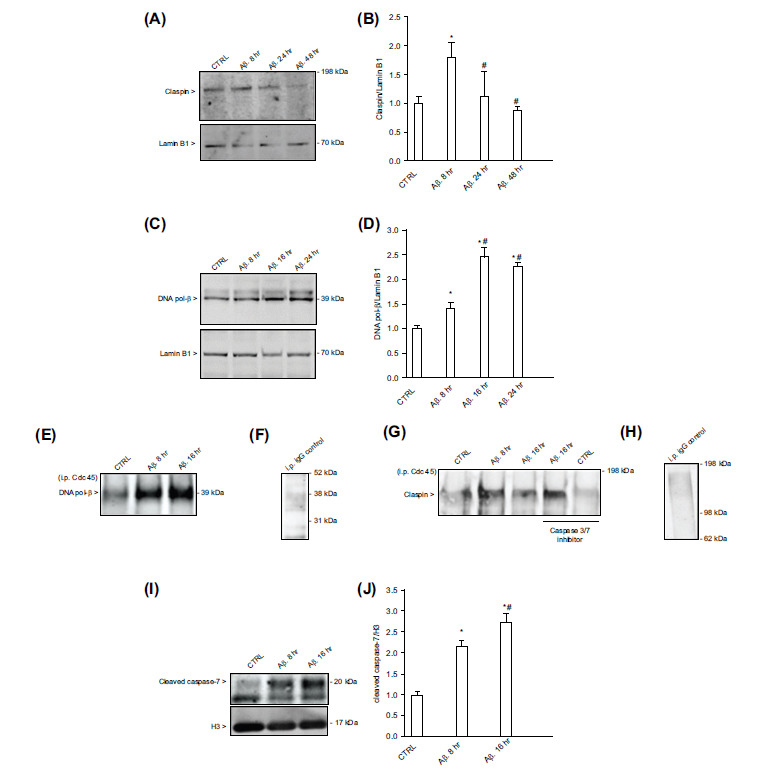
Claspin loading at replication forks in Aβ-treated neurons and its dependence on caspase-7 activity. Representative immunoblots of Claspin (**A**) and DNA pol-β (**C**) in total nuclear protein extracts from cultured cortical neurons challenged with 1 µM oligomeric Aβ_(1-42)_ for different times. Both in (**A**) and in (**C**), the Lamin B1 band is shown as a loading control. (**B**) Densitometric analysis of Claspin bands from four separate western blots. The Claspin/Lamin B1 ratio was normalized to 1 in controls. Bars represent the means ± SEM of 4 determinations. *p < 0.05 *vs*. CTRL (controls) and ^#^*p <* 0.05 *vs*. Aβ 8 hr. (**D**) Densitometric analysis of DNA pol-β bands from three separate western blots. The DNA pol-β/Lamin B1 ratio was normalized to 1 in controls. Bars represent the means ± SEM of 3 determinations. *p < 0.05 *vs*. CTRL (controls) and ^#^*p <* 0.05 *vs*. Aβ 8 hr. Immunoprecipitation of 1,000 bp-sized nucleoprotein fragments with anti-Cdc45 antibody, and blotting with anti- DNA pol-β antibody (**E**) or anti-Claspin antibody (**G**). Nucleoprotein fragments were obtained from neurons challenged with 1 µM oligomeric Aβ_(1-42)_ for 8 or 16 hr. Caspase-3/7 inhibitor (500 nM) was added where indicated. In (**F**) and (**H**), representative images of mock IP that were carried out on neuronal nucleoprotein fragments with the IgG isotype control of Cdc-45 before blotting with anti-DNA pol-β antibody (**F**) or anti-Claspin antibody (**H**). (**I**) Representative immunoblot of the large subunit (~20 kDa) of cleaved/active caspase-7 in nuclear protein extracts from cultured cortical neurons challenged with 1 µM oligomeric Aβ_(1-42)_ for 8 or 16 hrs. The histone H3 band is shown as loading control. **J**) Densitometric analysis of the cleaved caspase-7 bands from three separate western blots. The cleaved caspase-7/Lamin B1 ratio was normalized to 1 in controls. Bars represent the means ± SEM of 3 determinations. *p < 0.05 *vs*. CTRL (controls) and ^#^*p <* 0.05 *vs*. Aβ 8 hr.

Claspin is a conserved DNA binding protein that functions in replication checkpoints of mammalian cells as an adaptor protein between ATM/ATR and the downstream Chk-1 [13]. In extracts from Xenopus eggs, Claspin associates with incipient DNA replication forks and uses a conserved domain to interact with key replication and checkpoint proteins, including Cdc45 [11, 22]. Claspin was expressed in neuronal nuclear extracts, increased 8 hr after exposure to Aβ_(1-42)_ oligomers and gradually declined after 24 hr (Figs. **4A**, **B**). In comparison, DNA pol-β increased 8 hr after exposure to Aβ_(1-42)_ oligomers and reached a plateau between 16 and 24 hr after the Aβ challenge (Figs. **4C**, **D**). Then, we performed co-immunoprecipitation experiments on cross-linked nucleoprotein fragments at 8 and 16 hr after neuronal exposure to Aβ_(1-42)_ oligomers (*i.e*., an intermediate time between 8 and 24 hr, when apoptosis is generally triggered [6]). Cdc45, used as an index of licensed origins, was co-immunoprecipitated with both DNA pol-β and Claspin (Figs. **4E**, **4G**). However, while the levels of loaded DNA pol-β persisted between 8 and 16 hr following Aβ exposure, the levels of loaded Claspin decreased at 16 hr (Figs. **4E**, **4G**). It has been demonstrated that Claspin is cleaved by caspase-7 during the initiation of apoptosis in HeLa cells, where Claspin cleavage coincides with the inactivation of Chk-1 signaling [12]. Interestingly, the loss of Claspin occurring at the neuronal replication forks 16 hr after Aβ challenge was prevented by the presence of the caspase-3/7 inhibitor I (500 nM), indicating that it was due to caspase activity (Fig. **4G**). Accordingly, western blot analysis of neuronal nuclear extracts showed the presence of low levels of the large subunit (~20 kDa) of cleaved/active caspase-7 under basal conditions, which increased in response to Aβ_(1-42)_ oligomers in a time-dependent manner (Figs. **4I** and **J**).

### 3.3 Caspase-3/7 Inhibitor I held Neurons into the S Phase and Reduced Apoptosis

**Fig. (5):**
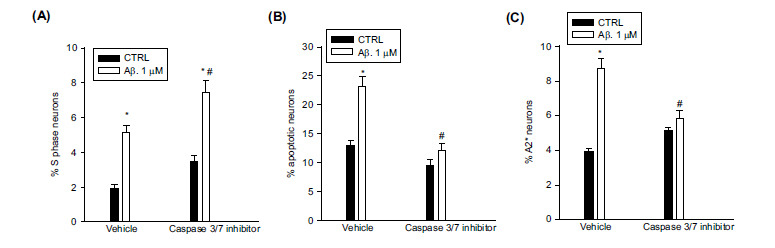
The caspase-3/7 inhibitor I held Aβ-treated neurons into the S phase and reduced apoptosis. Pure neuronal cultures were exposed to caspase-3/7 inhibitor (500 nM) for 1 h and then treated for 24 hr with 1 µM oligomeric Aβ_(1-42)_. S phase (**A**) and apoptotic (**B**) neurons were scored by cytofluorimetric analysis of PI-labeled samples. Values are means ± SEM of n = 17 from 5 independent experiments. *p < 0.05 *vs*. CTRL (control) and ^#^*p <* 0.05 *vs*. Aβ alone. (**C**) The caspase-3/7 inhibitor I reduced the population of cyclin A2^+^ neurons in Aβ-treated neurons. Neurons were immunostained with Cyclin A2 antibody and scored by flow cytometry. Values are means ± SEM of n = 4. *p < 0.05 *vs*. CTRL (control) and ^#^*p <* 0.05 *vs*. Aβ alone.

Having identified Claspin as a substrate for caspase 7, and assuming that Claspin degradation was responsible for the entry into apoptosis of replicating neurons, we assessed the effects of caspase-3/7 inhibitor I on cell cycle distribution profiles and death of neurons challenged with Aβ oligomers for 24 hr. According to our hypothesis, we found that the caspase-3/7 inhibitor I increased the percentage of S phase neurons while reducing the percentage of apoptotic neurons in response to Aβ oligomers (Figs. **5A**, **B**).

**Fig. (6):**
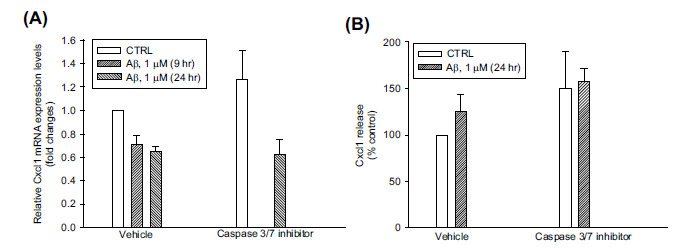
Lack of effect of Aβ oligomers on Cxcl1 mRNA expression and release in pure cortical neurons. Pure neuronal cultures were treated for 9 or 24 hr with 1 µM oligomeric Aβ_(1-42),_ both in the presence and in the absence of 500 nM caspase-3/7 inhibitor I. (**A**) Cxcl1 mRNA expression was examined by qRT-PCR. The abundance of mRNA was expressed relative to the abundance of GAPDH mRNA, as an internal control. qRT-PCR amplifications were performed in duplicate for each biological sample. Values are means ± SEM of 3-4 biological replicates. (**B**) The modulation of Cxcl1 release by Aβ treatment. Supernatants from pure cortical neurons challenged with Aβ for 24 hr, both in the presence and in the absence of 500 nM caspase-3/7 inhibitor I, were analyzed using a Cxcl1/Cinc-1 Quantikine ELISA Kit. Tests were performed in duplicate for each biological sample. Values are means ± SEM of 3-6 biological replicates. Cxcl1 release was expressed as the percent variation with respect to the production assessed in control neurons. Basal levels of Cxcl1 in the medium from control neurons were 16.34 ± 2.45 pg/ml (mean ± SEM). Neither in A nor in B did the caspase-3/7 inhibitor I influence Aβ effects.

We wondered about the fate of neurons rescued from apoptosis by caspase-3/7 inhibitor I. FACS analysis of neurons immunostained for cyclin A2, which is required for DNA replication and mitotic entry [23], showed that a 24 hr exposure to Aβ_(1-42)_ oligomers increased the percentage of cyclin A2^+^ neurons, in agreement with the presence of DNA replication in Aβ-challenged neurons. However, the pre-exposure to caspase-3/7 inhibitor I reduced the population of cyclin A2^+^ neurons (Fig. **5C**), despite the increase in surviving neurons (Fig. **5B**). Since cyclin A2 is quickly degraded as cells enter mitosis [24], caspase-3/7 inhibitor I possibly allowed Aβ-challenged neurons to survive until entry into mitosis. To get an idea of the extent to which these neurons were functional or dysfunctional, we addressed the mRNA expression and release of Cxcl1. Cxcl1 is one of the inflammatory chemokines that have been associated with the appearance of senescence in cycling neurons derived from C9orf72 ALS patients [14] and to tau hyperphosphorylation [25]. We found that Aβ_(1-42)_ oligomers did not increase the expression levels of Cxcl1 mRNA (Fig. **6A**) and did not increase significantly Cxcl1 release (Fig. **6B**), suggesting that neurons induced by Aβ oligomers to enter the S phase were not prone to assume an inflammatory phenotype. The addition of caspase-3/7 inhibitor I to Aβ, which delayed the entry into apoptosis of S phase neurons (Figs. **5A**, **B**), did not promote the onset of the inflammatory phenotype (Figs. **6A**, **B**).

### 3.4 A phosphopeptide Derived from the Chk-1-Binding Motif of Claspin Promoted Chk-1 Phosphorylation and Reduced Apoptosis

**Fig. (7):**
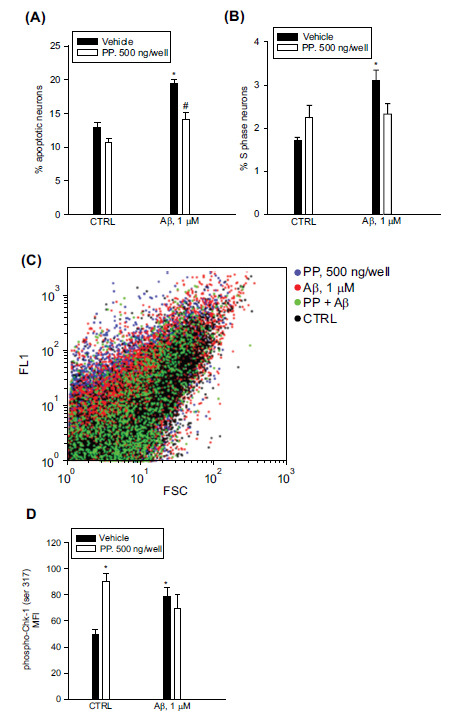
Transfection of PP, a phosphopeptide mimicking the Chk-1-recognition motif of Claspin, prevented apoptosis in Aβ-challenged neurons and promoted Chk-1 (ser 317) phosphorylation. Pure neuronal cultures were transfected with PP (500 ng/well) 1 hr before washing and adding 1 µM Aβ_(1-42)_ oligomers for 24 hr. Apoptotic (**A**) and S phase neurons (**B**) were scored by cytofluorimetric analysis of PI labeled samples. Values are means ± SEM of n = 4-5 for experimental condition. *p < 0.05 *vs*. CTRL (control) and ^#^*p <* 0.05 *vs*. Aβ alone. (**C**) Dot blot histogram (FSC versus FL1) of neurons stained with phospho-Chk-1 (ser 317) antibody. The histogram was obtained with the FCS Express 5 software. FSC refers to the forward-angle scatter parameter of cells, whereas FL1 refers to the green fluorescence of phospho-Chk-1 (ser 317). Note the higher FL1 values of the blue and red populations corresponding to PP-transfected neurons and Aβ-challenged neurons, respectively. (**D**) The median fluorescence intensity (MFI) of phospho-Chk-1 (ser 317) was determined by flow cytometry using the Flowing Software 2.5.1. Values are means ± SEM of n = 5-6 for experimental condition. *p < 0.05 *vs*. CTRL (control).

Studies carried out in Xenopus indicate that the Chk-1 recognizes a short, repeated, phosphorylated motif on the Chk-1-binding domain of Claspin [26]. By comparing the Chk-1-binding domains in Claspin homologues from different vertebrates, Clarke and Clarke designed a phosphopeptide (PP) to mimic this recognition motif. In nuclear extracts for HeLA cells, PP reduced the interaction between Claspin and Chk-1 and inhibited the phosphorylation of Chk-1 [17]. By using the Chariot transfection reagent, we transfected PP into cultured neurons to verify whether it was able to enhance Aβ toxicity by destroying the connecting bridge between Claspin and Chk-1. Chariot (also known as Pep-1 peptide) has the ability to localize to the nucleus [27], and it has been successfully used to deliver proteins and peptides into living neurons [28]. Preliminarily, the efficiency of the transfection was tested by delivering the β-gal positive control protein (1 µg/well), which entered about 40% of the neuronal population (Supplementary Fig. **S4**). PP transfection (500 ng/well) was carried out for 1 hr before washing and adding Aβ_(1-42)_ oligomers for 24 hr. PP did not affect basal neuronal apoptosis, and, to our surprise, it reduced Aβ-induced apoptosis rather than potentiating it (Fig. **7A**). No significant effect was observed on the Aβ-induced S phase (Fig. **7B**). Then, after treatment, we carried out a phospho-Chk-1 (ser 317) immunostaining in neurons harvested for flow cytometry (Fig. **7C**). We found that the median fluorescence intensity of phospho-Chk-1 (ser 317) was higher in PP-transfected neurons than in controls (Fig. **7D**), suggesting that PP was effective in mediating the phosphorylation of endogenous Chk-1. As expected, Aβ_(1-42)_ oligomers increased phospho-Chk-1 (ser 317) immunostaining, which was not significantly different in the combination PP + Aβ_(1-42)_ oligomers (Fig. **7D**). Hence, the transfected PP, mimicking the Chk-1-recognition motif of Claspin, was sufficient to promote Chk-1 (ser 317) phosphorylation with ensuing prevention of apoptosis in Aβ_(1-42)_-challenged neurons.

## 4 DISCUSSION

The resumption of the cell cycle by post-mitotic neurons is closely associated with apoptosis in several neurodegenerative conditions. Therefore, the mechanisms linking neuronal loss of quiescence to neuronal death are intensively investigated in the hope to discover new drug targets for neuroprotection. In search for these mechanisms, some conflicting reports with the general consensus have recently emerged.

Zhang and colleagues raised the issue that embryonic cortical neurons in cultures contain a subpopulation of cycling Nestin^+^ neuronal-like precursors that have been mistaken for post-mitotic neurons [29]. Since our cultures of pure cortical neurons, virtually devoid of GFAP^+^ cells, do not express Nestin, we are confident that no neuronal-like precursor has been mistaken for cortical neuron. Specifically, we used rat cortical neurons at 8 DIV, *i.e*. at a time in which fairly mature neuronal networks capable of spontaneous and evoked burst firing are described [30]. Different cultures contained from less than 1% up to just over 2% of S phase neurons, which could result from the spontaneous reentry into the cell cycle of post-mitotic neurons subjected to oxidative stress [31].

Ippati and colleagues have recently offered a different perspective on neuronal cell cycle re-entry by showing that neurons that highly express the S phase protein, geminin, are protected from Aβ toxicity [32]. It should be underlined that no DNA replication is observed under those experimental conditions, whereas others and we demonstrated that post-mitotic neurons die if they cross the G1/S transition [6, 33, 34] and, in the specific case of Aβ toxicity, we showed that DNA replication, carried out by DNA pol-β, is the trigger for death [7, 8].

A subtle difference between our previous works and the present one is that we have now used a low concentration of synthetic Aβ_1−42_ oligomers, instead of a high concentration (25 μM) of Aβ_25−35_ aggregates, as a neuronal challenger. Aβ_25−35_ is the short active fragment of full-length Aβ_1−42_ that is toxic soon after solubilization [6] and, compared to Aβ_1−42_ oligomers, is faster active by promoting the rapid unloading of DNA pol-β from neuronal replication forks and the subsequent triggering of the death signal [8]. However, synthetic Aβ_1−42_ oligomers show properties equivalent to those of soluble Aβ oligomers from the AD brain [15], and we preferred to use them even though their effects are less pronounced.

Intrigued by the hint that AD neurons maintain their DNA replication status over a long time, even years [5], we decided to investigate the ATM-ATR/Claspin/Chk-1 pathway, which, in proliferating cells, sets the threshold between DNA replication and death [11, 12]. In preliminary experiments, caffeine, which is a non-selective direct inhibitor of ATM kinase [20], increased Aβ-induced S phase and apoptosis in neurons. Afterwards, we used a cell-permeable small molecule to inhibit selectively the checkpoint kinases ATM and ATR, which share many substrates, including Chk-1, and monitor the status of DNA during cell cycle progression [35]. The ATM/ATR inhibitor we used, also known as CGK-733, failed to inhibit ATM/ATR kinase activity in human lung cancer cells [36]. However, additional studies suggest that this compound inhibits the ATM/ATR kinase in a variety of cell systems [37-40] and specifically inhibits the ATM kinase activity monitored by a reporter molecule in living cells and animals [41]. We found that the inhibition of the ATM/ATR kinase activity on its own did not affect neuronal survival, but it amplified Aβ-induced S phase and apoptosis by a DNA pol-β-dependent mechanism, in keeping with the hypothesis that a compromised DNA replication checkpoint could bring replicating neurons closer to death. Our experimental model mimics the AD brain condition, where signs of reduced ATM function and cell cycle activation coexist in vulnerable neurons, although the cause of the ATM loss remains unknown [19]. On the contrary, our evidence that the pharmacological inhibition of the ATM/ATR kinase *per se* did not affect the basal number of neurons entering the S phase differs from the demonstration that a partial genetic deletion of ATM is sufficient to re-activate cell cycle in some neurons from ATM^+/-^ mice [19]. Apparently, the transient pharmacological inhibition of ATM kinase in differentiated neurons does not recapitulate the ATM protein deficiency during embryogenesis.

ATM/ATR kinase and Chk-1 operate along the same pathway [21]; hence, it was not surprising that the pharmacological inhibition of Chk-1 had the same effects as the blockade of ATM/ATR kinase. Accordingly, we found that, in response to Aβ_(1-42)_ oligomers, Chk-1 was phosphorylated by ATM/ATR kinase at ser-317, an amino acid residue that is critical for Chk-1 activation [42]. However, we also found that total Chk-1 levels were increased independently of ATM/ATR kinase, perhaps because Chk-1 levels may be positively regulated by the transcription factor E2F as cells enter the S phase [43]. Once activated by phosphorylation, Chk-1 is generally required to inhibit Cdc25 phosphatases, which allow the activity of cyclin-dependent kinases [44], but several other proteins that are involved in DNA replication and repair have been recently identified as substrates of Chk-1 [45]. So far, we have not identified the direct target of Chk-1 that helps to prolong the cell cycle in neurons challenged with Aβ oligomers, which otherwise would succumb quickly to a p53-mediated apoptotic pathway [7].

In order to assess whether Claspin could be involved in the transmission of replication stress signals from ATM/ATR kinase to Chk-1, we investigated the presence of Claspin at the neuronal origins of replication identified by Cdc45. In neurons exposed to Aβ oligomers, Claspin was found to be present at the same early time of DNA pol-β, in agreement with its role in the initiation of DNA replication [8], but its expression diminished at a later time, generally coinciding with the triggering of apoptotic demise. Interestingly, the neuronal transfection of PP, a short phosphopeptide designed by Clark and Clarke [17] to mimic the Chk-1-binding motif of Claspin, was *per se* sufficient to mediate the phosphorylation/activation of Chk-1 and prevented Aβ-challenged neurons from entering apoptosis. This finding further indicated that Claspin mediated the activation of Chk-1 under basal and Aβ-induced replicative stress conditions, although it was unexpected. In fact, PP was thought to act as a competitor of Chk-1 binding to Claspin [17], thus enhancing Aβ-induced apoptosis. Although we cannot fully explain this discrepancy, our finding is in line with the demonstration by Lee and coworkers that the Chk-1-binding motif of Claspin is not a mere docking site, but rather has Chk-1-activating functions [22]. Unfortunately, we could not provide a correlation analysis between PP transfection and phospho-Chk-1 (ser 317) immunostaining at a single neuronal level as we decided not to use a fluorescent tag that could affect the delivery and the activity of the transfected PP. Along the same line of experimental evidence, a potent and membrane-permeable inhibitor of both caspase-3 and caspase-7, namely caspase-3/7 inhibitor I, rescued Claspin from degradation, kept Aβ-challenged neurons into the S phase, and reduced apoptosis. These data agree with the knowledge that caspase-7 cleaves Claspin during the initiation of apoptosis in HeLa cells, thus inactivating Chk-1 signaling [12]. Although caspase-3/7 inhibitor I was not selective for caspase-7, at least we found that low levels of active caspase-7 were expressed by cultured neurons and that these levels increased in response to Aβ oligomers in a time-dependent manner.

Noteworthy, the presence of active caspase-7 has been found in the brain of TgCRND8 mice, an early-onset AD mouse model where a chronic treatment with the caspase inhibitor, Q-VD-OPh, limited tau pathology [46]. Most relevant, a loss-of-function variant of the caspase-7 gene has been associated with a reduced incidence of AD in homozygous carriers of the high-risk APOE ε4 allele [47]. Hence, although caspase activation generally represents a terminal event in neurodegeneration, activation of caspase-7 might be a proximal event that sets the engagement of apoptosis in cycling neurons. We do not actually know how far Aβ-challenged neurons proceeded into the cycle when caspase-7 was inhibited. The evidence that the pre-exposure to caspase-3/7 inhibitor I reduced the population of cyclin A2^+^ neurons induced by Aβ oligomers, despite the increase in the total number of surviving neurons, suggested that caspase-3/7 inhibitor I allowed Aβ-challenged neurons to survive until entry into mitosis, when cyclin A2 is quickly degraded [24]. It must be said that cell cycle analysis of PI-stained neurons exposed to Aβ, and pretreated with caspase-3/7 inhibitor I, did not catch neuronal populations beyond the S phase; however, this could be due to the stochastic exit of neurons from the S phase.

Hence, caspase-7 inhibitors may provide an effective means of preventing the activation and execution of apoptosis in neurons that have resumed the cell cycle and have entered the S phase. So far, the functional changes of AD neurons, which survive in the hyperploid state, are unclear. Cultured cortical neurons, which were forced to hyperploidy by expressing SV40 large T antigen (TAg), exhibited a reduced synaptic activity; however, the model did not strictly represent the pathological situation of AD, because TAg-expressing neurons underwent a non-apoptotic cell death process [48].

The recent evidence that cycling ALS neurons exhibit a senescence-associated secretory phenotype (*i.e*., a pattern of inflammatory cytokines), which could be detrimental for neighboring cells, adds another level of complexity [14]. In our case, Aβ-challenged neurons, although replicating, did not increase the expression and release of Cxcl1, one of the endogenous inflammatory chemokines that were associated with the appearance of senescence in cycling ALS neurons [14] and that we have selected because it has been linked to tau hyperphosphorylation [25]. Most importantly, the addition of caspase-3/7 inhibitor I to Aβ, which lengthened the S phase of neurons and delayed apoptosis, did not seem to facilitate the onset of a neuronal inflammatory phenotype.

Overall, we suggest that Claspin degradation by intervening caspase-7 activation may precipitate the death of AD neurons engaged in DNA replication. Although the direct demonstration that the loss of Claspin is responsible for the demise of Aβ-challenged neurons is still lacking, we showed that a short phosphopeptide mimicking the Chk-1-binding motif of Claspin was able to prevent Aβ-challenged neurons from entering apoptosis.

## CONCLUSION

The present study demonstrates that the inhibition of the ATM/ATR signaling pathway amplified Aβ-induced S phase and apoptosis in cultured cortical neurons, thus indicating that the ATM-ATR/Claspin/Chk-1 pathway could function as a checkpoint between replication and death in cultured differentiated neurons. Accordingly, the inhibition of the degradation of the adaptor protein, Claspin, by a caspase-7 inhibitor or the transfection of a short phosphopeptide mimicking the Chk-1-binding motif of Claspin were associated with a lower number of apoptotic neurons following the challenge with Aβ. These findings assess the validity of therapeutics aimed at preventing Claspin degradation and sustaining Chk-1 activity in neurons that cycle towards apoptosis.

## Data Availability

The data that support the findings of this study are available from the corresponding author upon reasonable request.

## LIST OF ABBREVIATIONS

AD: Alzheimer’s Disease
ALS: Amyotrophic Lateral Sclerosis
Aβ: β-Amyloid
DNA pol-β: DNA Polymerase-β
ATM: Ataxia-Telangiectasia Mutated
ATR: Ataxia Telangiectasia and Rad3-Related
Chk-1: Checkpoint Kinase-1
E15: Embryonic Day 15
DIV: Days *In Vitro*
FACS: Fluorescence Activated Cell Sorting
PBS: Phosphate-Buffered Saline
BSA: Bovine Serum Albumin
RT: Room Temperature
SDS: Sodium Dodecyl Sulfate
PP: Phosphopeptide
β-gal: β-galactosidase
DDC: Dideoxycytidine
TAg: T Antigen
