# Supplementary material for: Molecular Connections between DNA Replication and Cell Death in β-Amyloid-Treated Neurons
Source: Curr Neuropharmacol. 2023 Jul 10;21(9):2006–18. doi: 10.2174/1570159X21666230404121903 (PMC10514525; doi:10.2174/1570159X21666230404121903)
Supplement: Supplementary file 1 [file CN-21-2006_SD1.pdf]

Supplementary Material

Molecular Connections between DNA Replication and Cell Death in β-Amyloid-Treated Neurons

Filippo Caraci[1,2], Annamaria Fidilio[2], Rosa Santangelo[1], Giuseppe Caruso[1], Maria Laura Giuffrida[3], Marianna Flora Tomasello[3], Ferdinando Nicoletti[4,5] and Agata Copani[1,3,*]

[1]Department of Drug and Health Sciences, University of Catania, Catania, Italy; [2]UOR of Neuropharmacology and Translational Neurosciences, Oasi Research Institute - IRCCS, Troina, Italy; [3]Institute of Crystallography, National Council of Research, Catania Unit, Catania, Italy; [4]Departments of Physiology and Pharmacology, University Sapienza of Rome, Rome, Italy; [5]IRCCS Neuromed, Pozzilli, Italy

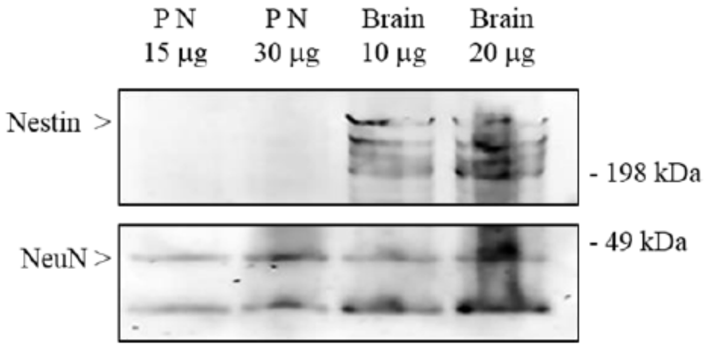

Supplementary Figure 1. Pure cortical neurons (P N) at 8 days *in vitro* express the neuronal marker, NeuN, and not the neuronal precursor marker, Nestin. A western blot image of P N protein extracts is shown. Protein extracts of newborn rat brains (Brain) were used as a positive control for nestin expression. The amount of loaded proteins in each lane is indicated.

1875-6190/XX $65.00+.00 © XXXX Bentham Science Publishers

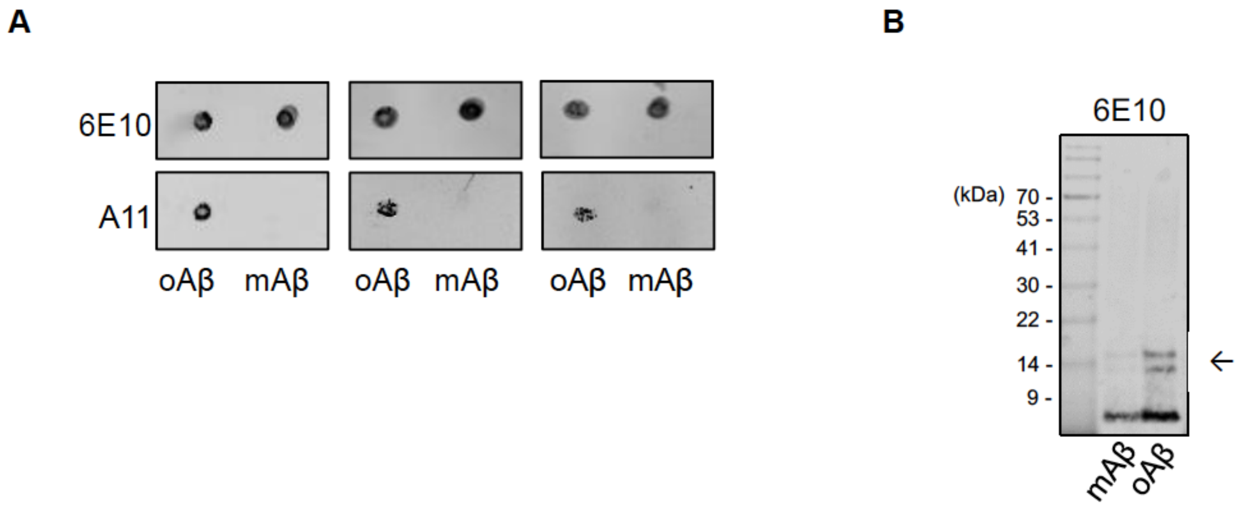

Supplementary Figure 2. Characterization of the oligomeric Aβ$_{(1\text{-}42)}$ preparation. A) Dot blot immunoassay using the antibody A11 detects Aβ$_{(1\text{-}42)}$ oligomers (oAβ) and not monomers (mAβ), whereas the use of the antibody 6E10 detects both oAβ and mAβ. Images from 3 independent preparations are shown. B) Western blot analysis of oAβ preparation, using the antibody 6E10, shows the presence of the 4-kDa monomers together with trimers and tetramers (arrow), which are almost indistinguishable in the mAβ preparation. 0.5 μg of either oAβ or mAβ were spotted in A, while 7.5 μg of either oAβ or mAβ were loaded in B.

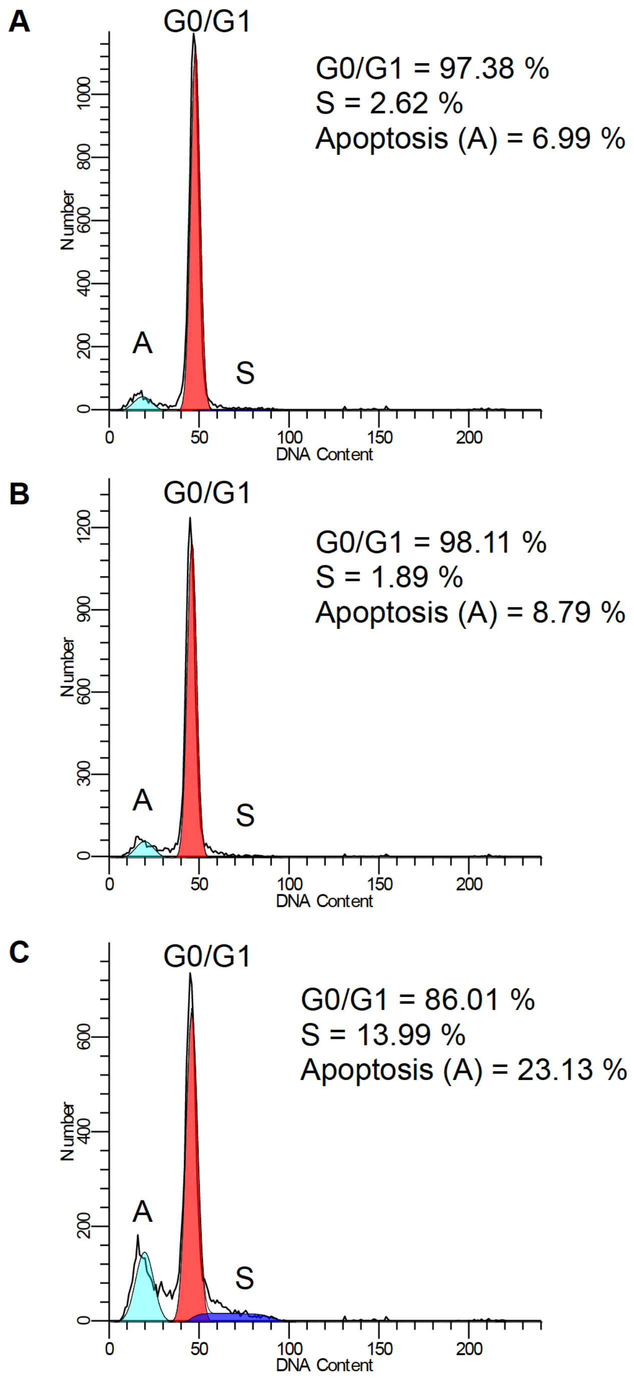

Supplementary Figure 3. Cell cycle distribution profiles of pure rat cortical neurons in culture. A) A typical cell cycle distribution profile of control neurons at 8 DIV where 97.38 % of the diploid population falls into the G0/G1 peak and hypoploid neurons, scored as apoptotic, are 6.99 % of the total population. **B)** A similar distribution is observed 18 hr after the addition of 0.1 μM A$\beta_{(1\text{-}42)}$ monomers. **C)** The cell cycle distribution profile changes markedly 18 hr after the addition of 1 μM A$\beta_{(1\text{-}42)}$ oligomers. The percentage of neurons in G0/G1 phase drops to 86.01 % and 13.99 % of neurons are in S phase (blue area under "S"). Concurrently, apoptotic neurons increase to 23.13 % of the total population. The 18 hr time point has been selected to make more visible the S phase peak, which usually decreases between 18 and 24 hours.

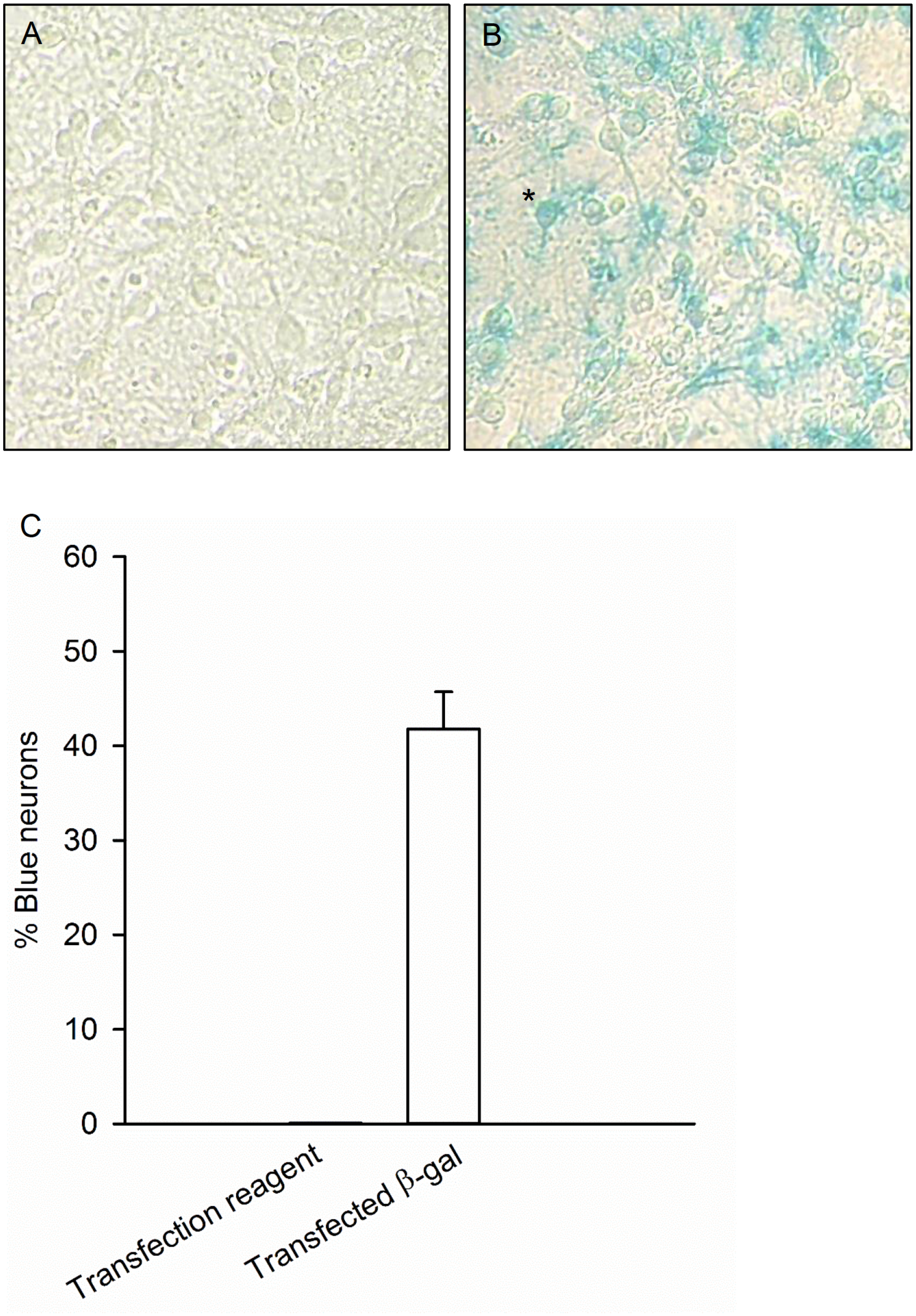

Supplementary Figure 4. Neuronal transfection of the β-gal positive control protein. Light microscopy images of neurons (320X magnification) that were exposed to the transfection reagent alone (**A**) or were tranfected with β-gal (1 μg/well) for 2 hr (**B**). In **B**, the asterisk indicates one of the neurons in which β-gal hydrolyses the substrate X-gal, thus producing a blue color. The percentage of blue neurons, scored from eight random fields from two different culture wells, is shown in **C**.

Supplementary Table 1. Inhibition of the ATM/ATR kinase by caffeine amplified Aβ-induced S phase and apoptosis in pure cortical neurons

Condition	% S phase neurons	% apoptotic neurons
Controls	2.375 ± 0.364	15.45 ± 1.598
Caffeine (2 mM)	6 ± 2.339	18.903 ± 1.093
$A\beta_{(1\text{-}42)}$ (1 μM)	9.075 ± 0.544*	27.92 ± 1.3*
Caffeine + $A\beta_{(1\text{-}42)}$	24.35 ± 4.25*#	34.052 ± 1.66*#

Pure neuronal cultures were exposed to the caffeine (2 mM) for 1 h and then treated for 24 hr with 1 μM oligomeric $A\beta_{(1\text{-}42)}$. S phase and apoptotic neurons were scored by cytofluorimetric analysis of PI labeled samples. Values are means ± SEM of 4 determinations. *$p<0.05$ vs. Controls and # $p<0.05$ vs. $A\beta_{(1\text{-}42)}$ alone.
